# Potential- and Buffer-Dependent Selectivity for the Conversion of CO_2_ to CO by a Cobalt Porphyrin-Peptide Electrocatalyst in Water

**DOI:** 10.1021/acscatal.2c03297

**Published:** 2022-11-16

**Authors:** Jose L. Alvarez-Hernandez, Alison A. Salamatian, Ji Won Han, Kara L. Bren

**Affiliations:** Department of Chemistry, University of Rochester, Rochester, New York14627-0216, United States

**Keywords:** biocatalyst, carbon dioxide reduction, cobalt porphyrin, electrocatalysis, overpotential

## Abstract

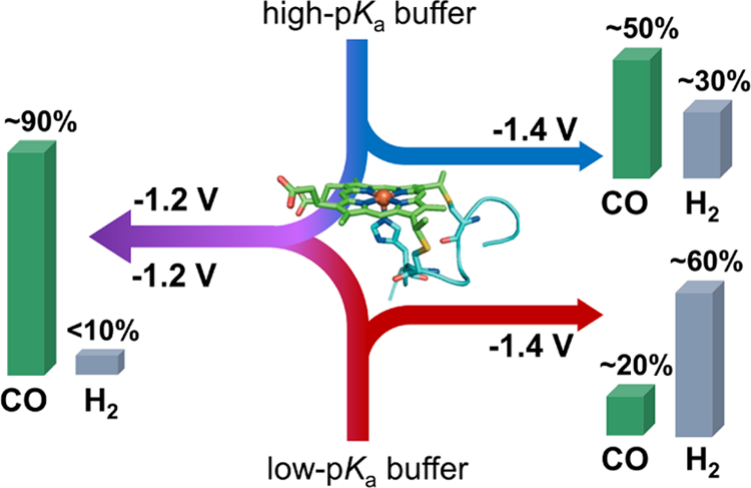

A semisynthetic electrocatalyst for carbon dioxide reduction to carbon monoxide in water is reported. Cobalt microperoxidase-11 (CoMP11-Ac) is shown to reduce CO_2_ to CO with a turnover number of up to 32,000 and a selectivity of up to 88:5 CO:H_2_. Higher selectivity for CO production is favored by a less cathodic applied potential and use of a higher p*K*_a_ buffer. A mechanistic hypothesis is presented in which avoiding the formation and protonation of a formal Co(I) species favors CO production. These results demonstrate how tuning reaction conditions impact reactivity toward CO_2_ reduction for a biocatalyst previously developed for H_2_ production.

## Introduction

Carbon dioxide (CO_2_) is an abundant and attractive feedstock for renewable fuels. Advances in catalysis are crucial for the development of systems for CO_2_ utilization,^1,2^ therefore attracting significant interest in the chemistry community.^2−6^ The inertness and stability of CO_2_ present both kinetic and thermodynamic barriers to its activation.^7^ The reduction of CO_2_ to any stable product is a multi-proton, multi-electron process (for example, see eq 1) with high activation energy, requiring effective catalysts to drive the process at acceptable rates.^8^ Molecular catalysts have proven successful in CO_2_ reduction reactions (CO_2_RR) and have enabled detailed mechanistic study,^5,8−16^ providing insight into the roles of both Brønsted^17−20^ and Lewis acids,^21,22^ as well as the coordination of electron transfer with both proton transfer and bond breaking and formation.^23^ Water is a desirable solvent to use for CO_2_ reduction,^5,24−30^ yet developing and understanding CO_2_RR catalysis in water brings several challenges: poor solubility of CO_2_ ([CO_2_] = 0.0383 M at 20 °C and 1 atm of CO_2_),^31^ pH-dependent equilibria among CO_2_ and its hydration products (H_2_CO_3_, HCO_3_^–^, and CO_3_^2–^), and competition from the hydrogen evolution reaction (HER; eq 2).
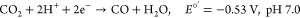
1
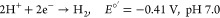
2

In electrocatalysis, the amount of energy beyond the thermodynamic requirements needed to drive a reaction at a given rate is described by the overpotential. Typically, lowering the overpotential for a given catalyst comes at the expense of slowing catalysis, with the log of the rate exhibiting a linear dependence on overpotential.^32−34^ The relationship between overpotential and catalyst selectivity is a less explored topic. Studies of potential-dependent product selectivity are reported for solid (nonmolecular) electrocatalysts,^35−37^ but studies of this nature for molecular catalysts are less common.^38−40^

**Figure 1:**
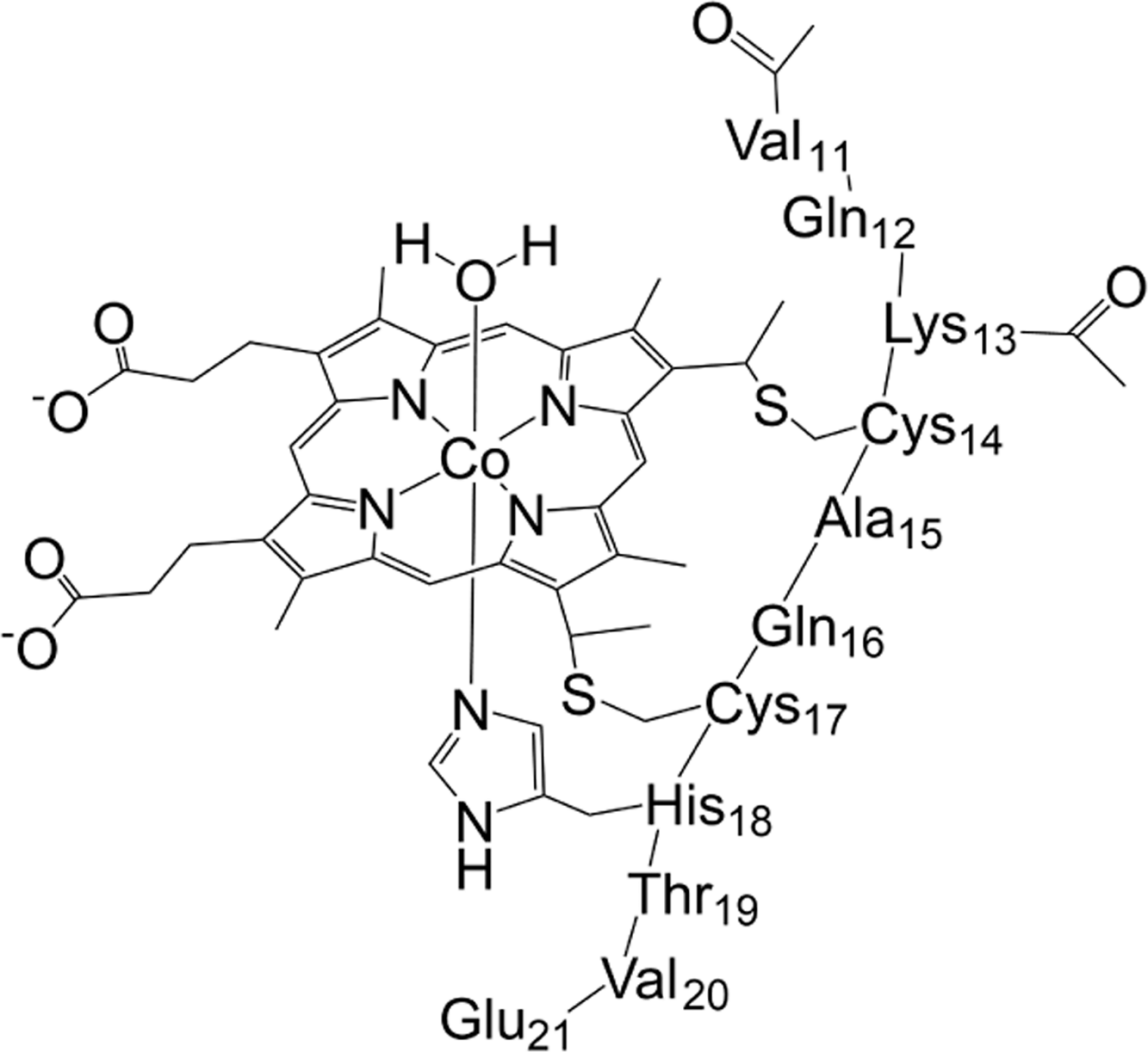
CoMP11-Ac. Reproduced with permission from ref (43). Copyright 2020, American Chemical Society.

This study reports on the effect of applied potential on selectivity for CO vs H_2_ production from CO_2_ in water by a biomolecular catalyst. We describe a cobalt porphyrin with a covalently attached peptide (CoMP11-Ac; Figure 1), previously described as a catalyst for HER,^41−43^ as an active and selective CO_2_-reduction electrocatalyst in water. CoMP11-Ac reaches a turnover number (TON) > 12,000 (at 2 h) for CO_2_ reduction to CO at an applied potential of −1.2 V (all potentials here are reported vs Ag/AgCl/KCl_(1M)_) with 85% faradaic yield. Our report is notable as a rare demonstration of the use of applied potential to control product selectivity for CO_2_RR by a molecular catalyst. Furthermore, a mechanistic proposal is put forward with the support of observed effects of potential, buffer acid p*K*_a_, and CO_2_ partial pressure on catalysis.

## Results and Discussion

### Effects of Potential

**Figure 2:**
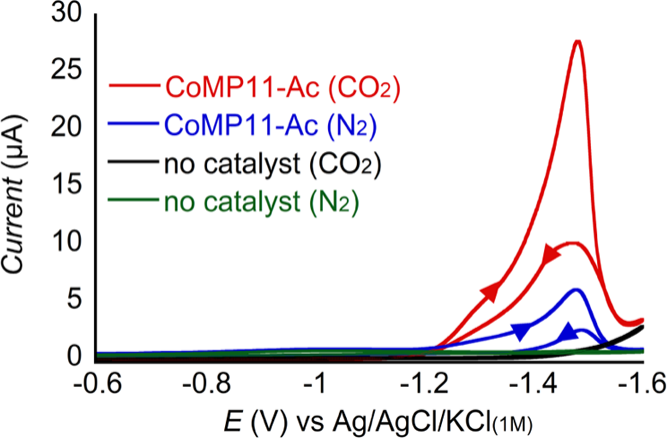
CVs of 1 μM CoMP11-Ac in 50 mM NaHCO_3_, 0.1 M KCl, pH 6.1 ± 0.1 at 100 mV/s, under 1 atm of the indicated gas. Arrows in the CV traces indicate the scanning direction.

The CO_2_RR activity of CoMP11-Ac in 50 mM NaHCO_3_ (p*K*_a1_ 6.4) solution saturated with CO_2_ was evaluated by cyclic voltammetry (CV) using a hanging mercury drop electrode, as shown in Figure 2. Dip-and-stir experiments^44^ reveal that CoMP11-Ac adsorbs to the mercury electrode, indicating that it behaves as an immobilized molecular catalyst (details in Figures S1–S4). A precedent for a system of this nature is that of Ni-cyclam, a CO_2_ reduction catalyst also adsorbed onto a mercury electrode.^45,46^ Importantly, the activity of particulate cobalt in this reaction is prevented by the use of a mercury electrode, as mercury amalgamates cobalt.^47−49^

The CVs of CoMP11-Ac, scanning from 0 to −1.6 V (and the opposite in the return scan), show no measurable current enhancement until −1.2 V when a broad first wave is observed, followed by a second feature of higher current with a half-wave potential (*E*_h_) of ∼ −1.4 V. The catalytic CV current for both features is significantly higher for CO_2_-saturated vs N_2_-saturated solutions at the same pH, suggesting that CoMP11-Ac may catalyze CO_2_RR. An inverted peak is also observed upon switching the scanning direction, which indicates that the catalyst is partially deactivated at low potentials and reactivated upon scanning anodically.^50^ This phenomenon has been described for other molecular catalysts based on cobalt,^51−53^ as well as other transition metals.^54−56^

**Figure 3:**
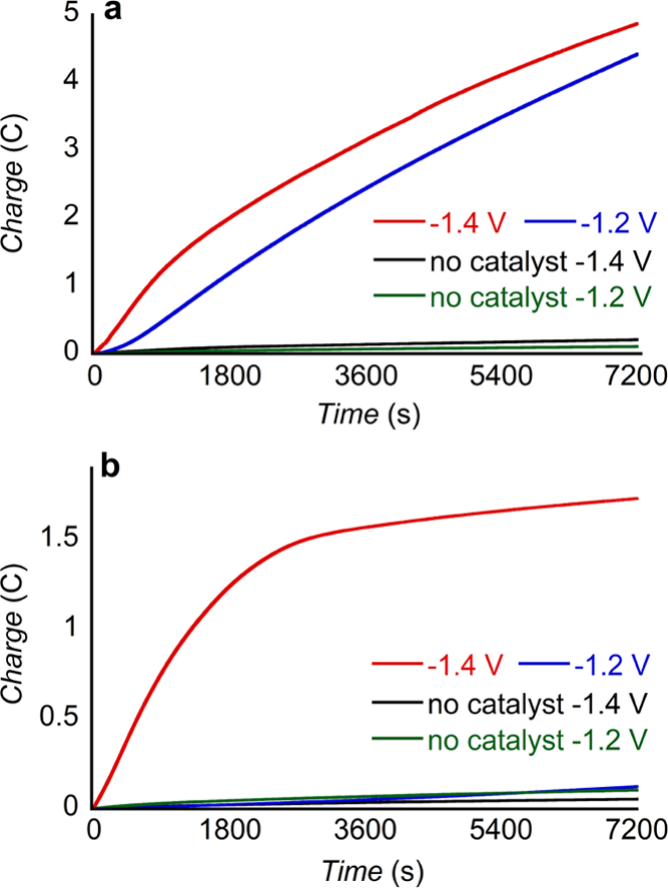
CPE experiments run in 0.5 M NaHCO_3_ and 1 M KCl; the concentration of CoMP11-Ac was 1 μM when present. CPE run in (a) CO_2_-saturated solution, pH = 6.5 ± 0.1 and (b) N_2_-saturated solution, pH = 8.7 ± 0.3. Samples were run under a pressure of 1 atm of the gas indicated (CO_2_ or N_2_). Potentials reported vs Ag/AgCl/KCl_(1M)_.

To further characterize the activity of this cobalt porphyrin-peptide toward CO_2_RR, we performed controlled potential electrolysis (CPE) experiments at both −1.2 and −1.4 V in 0.5 M NaHCO_3_ for 2 h in solutions purged with either CO_2_ or N_2_ and under 1 atm of the purging gas (Tables 1 and S1, and Figure 3). Under a CO_2_ atmosphere, the charge passed with CoMP11-Ac present is comparable at both applied potentials (Figure 3), yet the product distribution is rather different. The faradic efficiency for H_2_ (FE_(H_2_)_) decreases from 23% at −1.4 V to 4% at −1.2 V, while FE_(CO)_ increases from 61% at −1.4 V to 83% at −1.2 V. Furthermore, the turnover number (TON) for CO production measured after 2 h of CPE increases from 2500 at −1.4 V to 3300 at −1.2 V. In N_2_-saturated NaHCO_3_ solution at −1.4 V, CoMP11-Ac produces H_2_ with a 76% FE and CO with a 16% FE; CO arises from the reduction of the CO_2_ in equilibrium with NaHCO_3_ buffer.

**Table 1:** Summary of 2-h CPE Results for 1 μM CoMP11-Ac in 0.5 M NaHCO_3_^a^

	*E* (V)^b^	FE_(H_2_)_ (%)	FE_(CO)_ (%)	TON_(H_2_)_	TON_(CO)_	*Q*_T_ (C)
CO_2_	–1.4	23 ± 2	61 ± 4	1000 ± 200	2600 ± 400	4.0 ± 0.7
	–1.2	4 ± 1	84 ± 13	140 ± 40	3300 ± 100	3.7 ± 0.9
N_2_	–1.4	77 ± 5	15 ± 8	1200 ± 300	230 ± 80	1.4 ± 0.3
	–1.2	no above-background activity				

a Data corresponds to the average of at least three individual runs, and the errors correspond to the difference between the average and the replicate of greatest difference from the average. Activity is not reported if it did not exceed three times background in more than one replicate. The pH of the NaHCO_3_ solutions after purging with CO_2_ was 6.5 ± 0.1 and 8.7 ± 0.3 when purged with N_2_.

b Potentials reported vs Ag/AgCl/KCl_(1M)_.

There are a few reports of potential-dependent selectivity in molecular CO_2_RR catalysts.^38−40^ For example, in a study of a group of Pd complexes, those complexes with more negative reduction potentials favor protonation of the metal to form a hydride (proposed to primarily lead to HER), whereas the complexes with less negative potentials favor protonation of Pd-coordinated CO_2,_ yielding CO.^39^ In a more recent study of Pd molecular catalysts, the authors sought to improve the selectivity for CO_2_-to-CO conversion by increasing the overpotential of HER, which was achieved by installing proximal cations in the second sphere of the catalyst.^40^

**Scheme 1:**
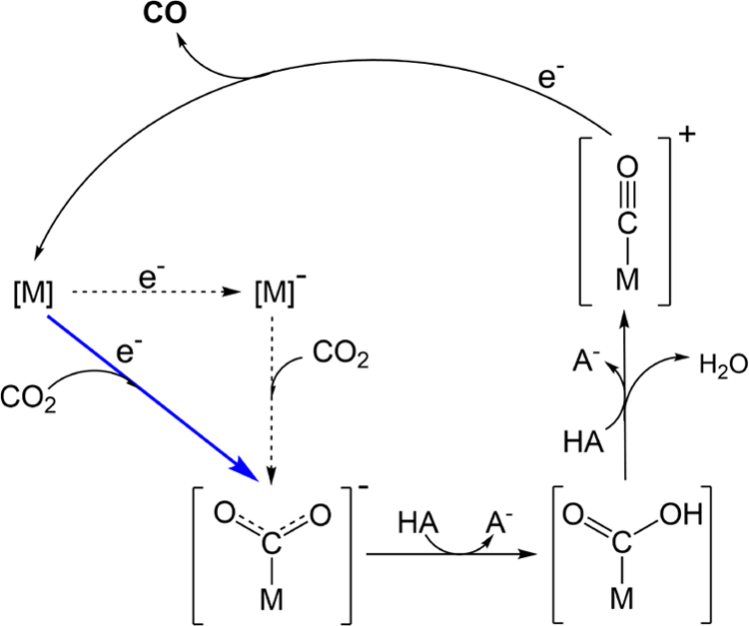
CO_2_-to-CO Catalysis Mechanism Proposed to Operate at −1.2 V vs Ag/AgCl/KCl_(1M)_ The diagonal blue arrow shows the CO_2_-addition-coupled electron transfer proposed to initiate catalysis; M/M^–^ corresponds to the formal Co(II/I) reduction.

In our case, we propose that the distinct behavior of CoMP11-Ac arises from a dependence of the CO_2_RR catalytic mechanism upon the applied potential. Because the *E*_Co(II/I)_ of CoMP11-Ac is estimated to be −1.42 V,^43^ the catalytic reduction of CO_2_ to CO at −1.2 V must originate from a different catalysis-initiating redox event. We propose that CO_2_ binding to the catalyst is coupled to the electroreduction of the catalyst (Scheme 1). This phenomenon where the formation or cleavage of a bond between heavy (non-hydrogen) atoms is coupled to electron and/or proton transfer has been invoked in electrochemical systems before.^23^ One example comes from the analysis of the rate-limiting O–O bond cleavage in the electrochemical reduction of aliphatic peroxides. When an all-concerted (coupling of the bond cleavage to both electron and proton transfer) pathway is at play, the CV feature associated with the electrochemically driven O–O bond cleavage was found to be at significantly less negative potential than when a stepwise mechanism is favored.^57^ In another example, an intermolecular concerted proton–electron transfer bond cleavage was also found to be the rate-determining step in the catalytic reduction of CO_2_ to CO by electrogenerated Fe(0) porphyrins in an aprotic solvent.^58^ Finally, the catalytic electroreduction of alkyl cobalt porphyrins is an example where carbon–metal bond breaking/formation and proton transfer are proposed to be concerted.^59^

Considering CO_2_ reduction by CoMP11-Ac, if a molecule of CO_2_ is appropriately prepositioned near the catalyst active site, it could bind the metal center in a manner concerted with electron transfer from the electrode to the Co(II) species ([M] in Scheme 1). Concerted pathways have the advantage of avoiding high-energy intermediates invoked in stepwise pathways.^23,60−64^ However, this advantage can be counterbalanced by other kinetic penalties. This is particularly likely in reactions that involve the breaking or formation of bonds between heavy atoms.^23^ The low CV current at −1.2 V, relative to the feature at −1.4 V, may be due to this additional kinetic expenditure. Prepositioning the CO_2_ molecule prior to binding to the metal center may be enabled or enhanced by conformational changes occurring upon adsorption of the catalyst on the mercury electrode. Similar effects have been found to account for the enhanced CO_2_RR activity of Ni-cyclam using a mercury working electrode. Adsorption of Ni(cyclam) onto the mercury electrode is proposed to cause a flattening of the ligand, leading to enhanced CO desorption kinetics (often the rate-determining step in CO_2_ reduction to CO by molecular catalysts) and diminished catalyst deactivation via CO poisoning.^45,46,65−67^

**Scheme 2:**
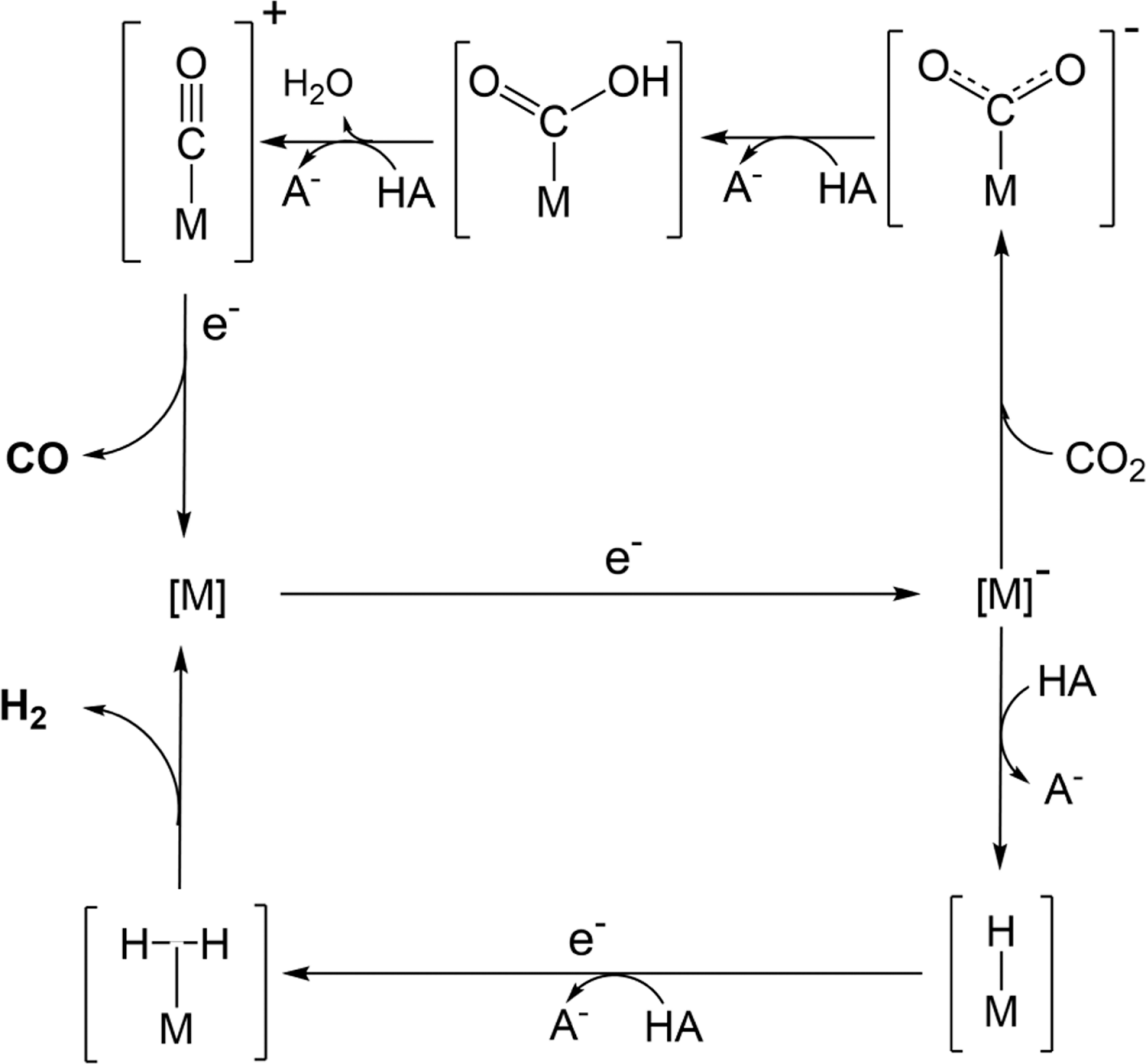
CO_2_-to-CO and HER Catalysis Mechanisms Proposed to Operate at −1.4 V vs Ag/AgCl/KCl_(1M)_ M/M^–^ Corresponds to the Formal Co(II/I) Reduction that Initiates Catalysis.

The more cathodic CV feature (at *E*_h_ = −1.4 V) develops at a potential near the Co(II/I) couple of CoMP11-Ac, suggesting that the dominant reaction mechanism at −1.4 V is initiated by the Co(II/I) reduction of the catalyst. Once the formal Co(I) species is formed, either CO_2_ addition or proton transfer from a proton donor HA to the catalyst may occur. Consequently, both CO_2_-to-CO and H_2_-evolution catalysis take place, resulting in lower selectivity for CO_2_ reduction at this more cathodic potential (Scheme 2).

### Effects of CO_2_ Partial Pressure (*P*_CO_2__)

In the mechanism outlined in Scheme 1 and proposed to be at play at −1.2 V, the catalysis-initiating redox event would entail a Nernstian equilibrium between Co(II)MP11-Ac (M in Scheme 1) and the CO_2_-bound one-electron reduced species, as depicted in eq 3.
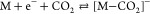
3Based on the Nernst equation for this process, we expect the half-wave potential (*E*_h_) to shift anodically with increasing partial pressure of CO_2_ (*P*_CO_2__) with a slope of 59.2 mV/decade, as shown in eqs 4 and 5. In these equations, *n* represents the number of electrons transferred (i.e., 1) and *E*°′ corresponds to the thermodynamic potential under standard conditions.
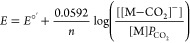
4
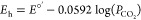
5

The CV feature near −1.2 V does not show a clear peak, hindering our ability to accurately determine *E*_h_. Instead, we define *E*_*i*_ as the potential at which a constant current of 1.5 μA is reached. eq 5 can be then rewritten in terms of *E*_*i*_ to obtain eq 6. (Please note that with this approximation, the *E*°′ term loses any physical meaning.) This approach of using the potential at which a constant current is reached has been employed as a proxy for *E*_h_ when non-ideal voltammograms are encountered.^68^
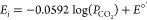
6

**Figure 4:**
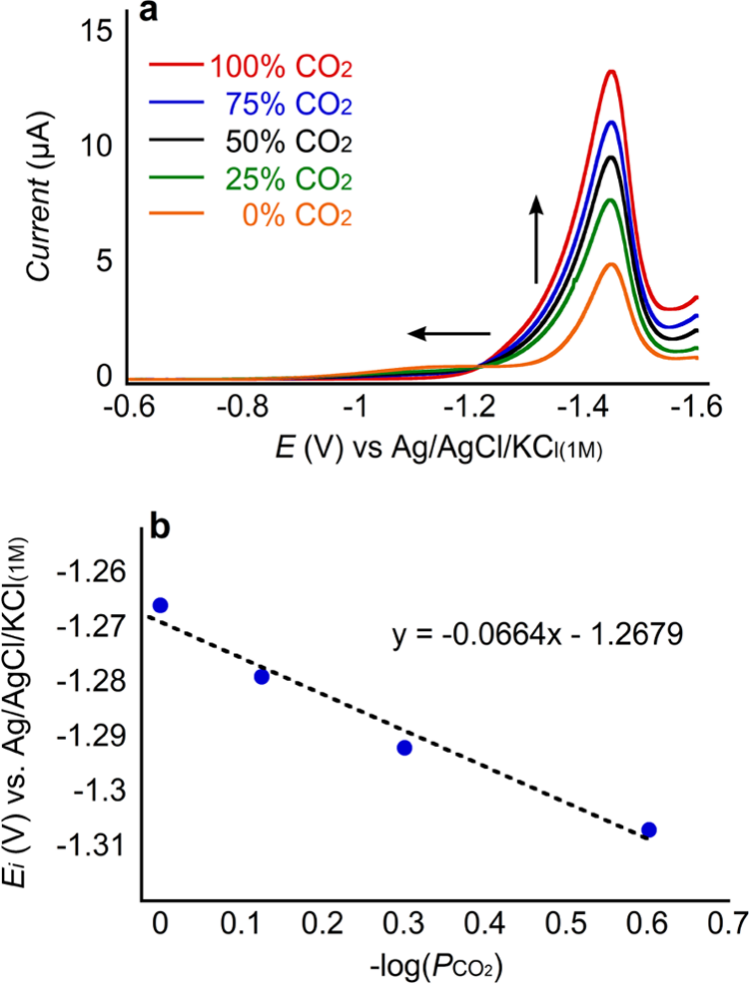
(a) Linear sweep voltammograms of 1 μM CoMP11-Ac in 50 mM CAPS, 0.1 M KCl, pH 6.0 ± 0.2 at 100 mV/s under different *P*_CO_2__; the arrows indicate the direction of increasing *P*_CO_2__. (b) Plot of *E_i_* vs −log(*P*_CO_2__) showing a Nernstian slope of ∼66 mV/decade.

We apply eq 6 to the voltammograms of CoMP11-Ac collected under different *P*_CO_2__ (Figures 4a and S5) achieved using mixtures of CO_2_ and N_2_ with different known compositions. To avoid deviations between *P*_CO_2__ and the concentration of CO_2_ in solution, we avoided the use of NaHCO_3_ as a buffer and instead used 3-(cyclohexylamino)-1-propanesulfonic acid (CAPS p*K*_a_ 10.4); more information regarding the effects of buffers is provided in the next section. In Figure 4a, we can see that as *P*_CO_2__ increases, the onset potential shifts anodically. A plot of *E*_*i*_ vs −log(*P*_CO_2__) (Figure 4b) shows a slope of ∼66 mV, supporting the proposal that the binding of CO_2_ is coupled to the one-electron reduction of the catalyst, as outlined in Scheme 1.

### Effects of Proton Donor

**Figure 5:**
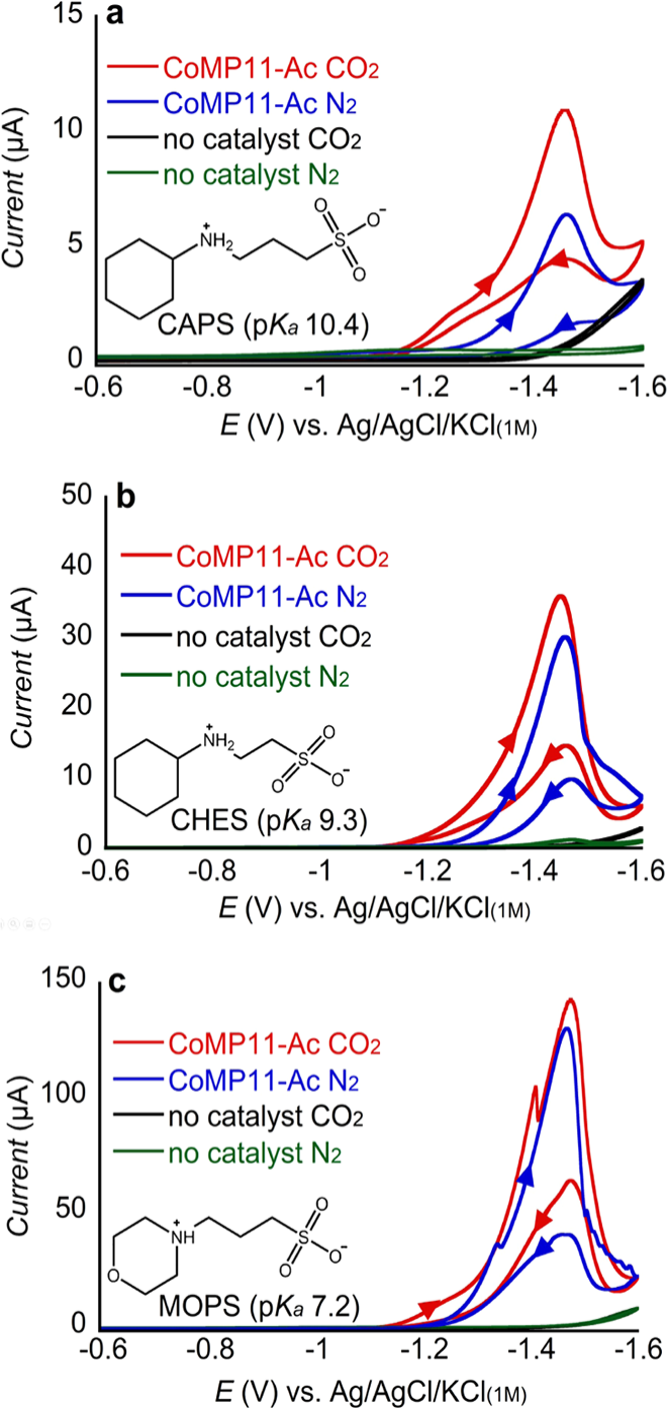
CVs of 1 μM CoMP11-Ac in (a) 50 mM CAPS, pH 5.3 ± 0.1; (b) 50 mM CHES, pH 5.9 ± 0.1; and (c) 50 mM MOPS, pH 5.9 ± 0.1. For all CVs, [KCl] = 0.1 M and scan rate = 100 mV/s. Arrows in the CV traces indicate the scanning direction.

To further test our mechanistic proposals, we varied the p*K*_a_ of the proton donor HA, which under our experimental conditions, we anticipate being the conjugate acid form of the buffer.^43,44,69^ It has been reported that the p*K*_a_ of the proton donor has a large impact on CO_2_-reduction catalysis. Relatively strong Brønsted acids lead to fast metal hydride formation and subsequent protonation to yield H_2_, as shown in the lower portion of Scheme 2; to minimize this undesirable pathway, weak Brønsted acids are preferred regardless of whether the catalyst operates in an aprotic or protic solvent.^3,5,17,70^ A particularly relevant example is the case of a water-soluble iron-porphyrin catalyst that was shown to evolve only H_2_ when using formate (p*K*_a_ 3.7) buffer, while an equimolar mixture of CO and H_2_ was obtained in phosphate-buffered solution (p*K*_a_ 7.2, H_2_PO_4_^–^).^30^ In the case of CoMP11-Ac, we have previously reported that the rate of HER in water decreases with increasing buffer p*K*_a_ due to a slower proton transfer from the buffer acid donor to the formal Co(I) and that such proton transfer to the formal Co(I) species is rate-limiting for buffers of p*K*_a_ > 7.7.^43^ We have also found that the sterics of the proton donor species impact the catalytic CV current arising from HER catalyzed by a cobalt porphyrin mini enzyme in water.^69^ When exploring the effects of buffer properties on the CO_2_RR of a Ni(cyclam) electrocatalyst in water, the authors concluded that charge density (i.e., charge and size) of the buffer acid species was the main factor impacting the catalytic activity.^71^ With these precedents in mind, we chose to study the CO_2_RR of CoMP11-Ac in the presence of three structurally related buffers as proton donors with different p*K*_a_ values: 3-(cyclohexylamino)-1-propanesulfonic acid (CAPS, p*K*_a_ 10.4), 3-(cyclohexylamino)-1-ethanesulfonic acid (CHES, p*K*_a_ 9.3), and 3-morpholinopropane-1-sulfonic acid (MOPS, p*K*_a_ 7.2; structures shown in Figure 5).

The CVs of CoMP11-Ac in CO_2_-saturated solutions containing either CAPS, CHES, or MOPS (Figure 5) exhibit two features, one that peaks around −1.5 V, present also in N_2_-saturated solution, and a more anodic wave that starts developing near −1.2 V, not present under N_2_. When the weakest Brønsted acid CAPS is present, the addition of CO_2_ leads to a significant enhancement in the catalytic CV current (Figure 5a); as the p*K*_a_ of the buffer acid decreases, the enhancement seen under CO_2_ relative to N_2_ becomes less pronounced. For MOPS solutions, the catalytic peak current seen for CoMP11-Ac is similar under both CO_2_ and N_2_ (Figure 5c). The lower p*K*_a_ of MOPS is proposed to facilitate proton transfer to the catalyst,^43^ yielding a higher CV current (faster catalysis) under both CO_2_ and N_2_. This result indicates possible enhancement of H_2_ and CO evolution, both of which are impacted by the availability of protons. The catalytic peak currents seen for CoMP11-Ac decrease as the p*K*_a_ of the buffer present increases. We attribute this trend to the lower acidity of the buffer acid species (i.e., higher p*K*_a_) disfavoring the transfer of protons, thereby slowing catalysis.

To assess whether product distribution is sensitive to the proton donor p*K*_a_ and the applied potential, we performed CPE experiments at both −1.4 and −1.2 V in solutions containing CAPS, CHES, or MOPS buffers; the results are summarized in Table 2 (see Tables S2–S4 for information on individual runs). For the CPE experiments at −1.4 V under N_2_ (Figures S6–S8), the total charge passed in the presence of MOPS buffer is significantly higher than in CHES and CAPS. The lower p*K*_a_ of the conjugate acid form of MOPS allows for more evolved H_2_, leading to a higher charge buildup, consistent with the trends seen in CV above as well as prior work.^43^ At −1.4 V in all CAPS, CHES, and MOPS, H_2_ is the sole product detected under N_2_, with respective FE values of 83, 92, and 92%. The TON for H_2_ is over 40-fold higher for catalysis in the presence of MOPS compared to CAPS. Overall, the charge passed decreases with increasing buffer acid p*K*_a_, a finding that is consistent with previous studies of buffer effects on HER by CoMP11-Ac,^43^ as well as with similar observations made for other catalysts working in both aqueous and aprotic solvents.^19,44,69,72−75^

**Table 2:** Summary of 2-h CPE Results for 1 μM CoMP11-Ac in 0.5 M of the Specified Buffer^a^

	buffer	*E* (V)^b^	FE_(H_2_)_ (%)	FE_(CO)_ (%)	TON_(H_2_)_	TON_(CO)_	*Q*_T_ (C)
CO_2_	CAPS (p*K*_a_ 10.4)	–1.4	29 ± 6	48 ± 10	280 ± 10	470 ± 10	0.9 ± 0.2
		–1.2	5 ± 1	88 ± 11	80 ± 20	1500 ± 300	1.7 ± 0.6
	CHES (p*K*_a_ 9.3)	–1.4	43 ± 9	57 ± 4	940 ± 30	1300 ± 300	2.2 ± 0.3
		–1.2	6 ± 1	81 ± 2	250 ± 30	3500 ± 300	4.2 ± 0.4
	MOPS (p*K*_a_ 7.2)	–1.4	63 ± 13	21 ± 5	4100 ± 500	1400 ± 500	6.4 ± 0.8
		–1.2	8 ± 2	85 ± 2	1200 ± 100	12,000 ± 1000	14.1 ± 1.4
N_2_	CAPS (p*K*_a_ 10.4)	–1.4	83 ± 16	∼0	500 ± 90	∼0	0.6 ± 0.2
		–1.2	no above-background activity				
	CHES (p*K*_a_ 9.3)	–1.4	92 ± 11	∼0	2800 ± 100	∼0	3.0 ± 1.1
		–1.2	67 ± 5	∼0	590 ± 100	∼0	0.8 (0.1)
	MOPS (p*K*_a_ 7.2)	–1.4	92 ± 6	∼0	23,000 ± 2000	∼0	24.9 ± 4.8
		–1.2	98 ± 3	∼0	5000 ± 900	∼0	4.7 ± 0.6

a Data shown corresponds to the average of at least three individual runs, and the errors correspond to the difference between the average and the replicate with the greatest difference from the average. Activity is not reported if it did not exceed three times background in more than one replicate. The pH of all MOPS, CHES, and CAPS solutions after purging with CO_2_ was 6.5 ± 0.2, and 7.2 ± 0.2 when purged with N_2_.

b Potentials reported vs Ag/AgCl/KCl_(1M)_.

For CPE experiments on CoMP11-Ac conducted at −1.4 V under CO_2_ (Figures S6–S8 and Tables S2–S4), both CO and H_2_ are produced with appreciable yields for all three buffers. In CAPS, the FEs for CO and H_2_ are 48 and 29%, respectively, while in MOPS, these quantities are 21 and 63%. Thus, the p*K*_a_ of the buffer is found to impact the product distribution at −1.4 V, with the lowest-p*K*_a_ buffer MOPS favoring H_2_ formation the most. This finding supports the proposed mechanism (Scheme 2) and is consistent with other observations on CO_2_RR in water.^6,17,18,30,53,55,76,77^ We propose that the stronger the acid, the more rapidly the Co(I) species is protonated, enhancing the generation of H_2_. Weaker acids (CAPS and CHES) protonate this species more slowly, allowing CO_2_ binding to the formal Co(I) active species and leading to more conversion of CO_2_ to CO. This model is consistent with previous work on CoMP11-Ac, in which more acidic buffers were found to promote fast HER catalysis and were proposed to protonate the formal Co(I) species more rapidly.^43^

When CPEs of CoMP11-Ac are carried out at −1.2 V under N_2_ in CAPS and CHES, activity is low, being comparable to the background in CAPS and barely above background for CHES (Figures S6–S8 and Tables S2–S4). In MOPS, at −1.2 V, H_2_ is the only product and is detected with 98% FE and TON of 4,900 after 2 hours. This result indicates that −1.2 V is too anodic relative to *E*_(Co(II/I))_ to support HER activity unless a relatively acidic proton source (here, MOPS) is present. Previous work on HER by CoMP11-Ac showed that the presence of an acidic proton donor (p*K*_a_ < 7.7) gives rise to a kinetic shift in the CV, allowing catalysis to occur at −1.2 V.^43^ Results under CO_2_ reveal a sharp contrast. For CPEs of CoMP11-Ac at −1.2 V under CO_2_, the overall activity is significantly higher than under N_2_ (Figures S6–S8). FE_(CO)_ is nearly the same for CoMP11-Ac in all three buffers: CAPS (88%), CHES (81%), and MOPS (85%), and FE(H_2_) also is nearly the same in CAPS (5%), CHES (6%), and MOPS (8%) (Tables 2 and S2–S4). The insensitivity of the product distribution at −1.2 V to buffer p*K*_a_ supports our proposal that in the presence of CO_2_, catalysis is initiated by CO_2_ binding coupled to catalyst reduction, which avoids the accumulation of a formal Co(I) species, leading to almost exclusive CO formation regardless of the acidic strength of the proton donor (Scheme 1). In other words, the selectivity-determining step precedes any proton transfer from the buffer acids, favoring the formation of CO irrespective of the proton donor p*K*_a_. Also worth highlighting is the TON_(CO)_ of 12,000 achieved at −1.2 V after a 2-h CPE in MOPS under CO_2_, which compares well with other molecular electrocatalysts operating in water.^78−82^

An interesting trend seen in the CPEs of CoMP11-Ac in the presence of CO_2_ in all four buffers (CAPS, CHES, MOPS, and NaHCO_3_) is that the catalyst is not only more selective for CO_2_ reduction at the less cathodic potential of −1.2 V but also exhibits similar or higher TON_(CO)_ (Tables 1 and 2). We have previously reported that CoMP11-Ac experiences partial deactivation during electrocatalytic HER.^41^ This is consistent with the lower FE seen in CPE at the more negative potential (−1.4 V) and with the shape of the CV in which the current rapidly drops after reaching its maximum value between −1.4 and −1.5 V, as well as the inverse peak feature seen in the return scan, which is consistent with reactivation.^50^ We propose that enhanced catalyst deactivation is responsible, at least in part, for the lower total charge passed and overall FE at −1.4 V, (particularly when compared to −1.2 V under CO_2_). The coupled mechanism outlined in Scheme 1 would allow for CO_2_ reduction catalysis to occur at potentials at which catalyst deactivation is minimal, yielding the higher charge passed for CoMP11-Ac at −1.2 V under CO_2_. Indeed, the CPE traces of CoMP11-Ac at −1.2 V after 2 hours remain linear, indicating that the catalyst is still active (Figures S6–S8). Furthermore, CoMP11-Ac under CO_2_ in CAPS displays minimal deviation from linearity in the charge vs time CPE trace in a 24-h CPE at −1.2 V, yielding a TON_(CO)_ of 9300. The 24-h CPE of CoMP11-Ac under CO_2_ in MOPS reveals some loss of activity after ∼6 h, as the CPE trace levels off, yet this more acidic proton donor yields a TON_(CO)_ of 32,000 in 24 hs (Table S5 and Figures S9–S13).

To determine whether the enhanced catalyst deactivation at −1.4 V is responsible for the lower selectivity for CO at this potential, we performed CPE experiments on CoMP11-Ac under CO_2_ at −1.4 V for 24 hours (Figure S11 and Table S5) and compared FE_(H_2_)_ and FE_(CO)_ to the results obtained after the 2-hour CPE under otherwise identical conditions (Tables 2 and S2). The overall FE is lower at 24 h (69%), as expected for a longer bulk electrolysis experiment (attributed to more catalyst degradation), but FE_(CO)_ is similar at 24 h (58 ± 6%) and 2 h (48 ± 10%). Interestingly, FE_(H_2_)_ is lower at 24 h (11 ± 6%) vs 2 h (29 ± 6%), which suggests that the CoMP11-Ac deactivation product is not a more active HER catalyst. Instead, this data suggests that the deactivation product may be generated within the HER mechanism of CoMP11-Ac.

## Conclusions

CoMP11-Ac catalyzes the reduction of CO_2_ to CO in water with FE_(CO)_ up to 88%, with better selectivity at −1.2 V compared to −1.4 V. The high faradic efficiency for CO production seen in CPE at −1.2 V is proposed to originate from a distinct mechanism initiated by CO_2_ addition coupled to the reduction of the catalyst, avoiding accumulation of a formal Co(I) species. The lower selectivity found at −1.4 V is proposed to arise from the Co(II/I) reduction initiating catalysis, as the formal Co(I) species can undergo either CO_2_ addition or protonation, where the latter enables HER. Altogether, at the lower applied overpotential, CoMP11-Ac shows higher selectivity toward CO_2_-to-CO conversion as well as enhanced catalyst longevity. These results demonstrate how applied potential and proton donor p*K*_a_ act together to determine catalyst selectivity. An implication is that these factors may contribute to system selectivity in complex ways, requiring codesign when developing and optimizing catalytic systems.

## Supporting Information Available

Experimental details, additional electrochemical data, tables of CPE results (Tables S1–S5) (Figures S1–S19) (PDF)
