# Supplementary material for: Potential- and Buffer-Dependent Selectivity for the Conversion of CO2 to CO by a Cobalt Porphyrin-Peptide Electrocatalyst in Water
Source: ACS Catal. 2022 Nov 16;12(23):14689–97. doi: 10.1021/acscatal.2c03297 (PMC9724230; doi:10.1021/acscatal.2c03297)
Supplement: Supplementary file 1 — cs2c03297_si_001.pdf [file cs2c03297_si_001.pdf]

Supporting Information

Potential- and Buffer-Dependent Selectivity for the Conversion of CO_2 to CO by a Cobalt Porphyrin-Peptide Electrocatalyst in Water

Jose L. Alvarez-Hernandez†, Alison A. Salamatian†, Ji Won Han, and Kara L. Bren*

Department of Chemistry, University of Rochester. Rochester, NY 14627-0216, United States

†Authors contributed equally

*email: bren@chem.rochester.edu

Table of Contents

SI-1 Experimental Details. 2

SI-2 Dip and Stir (D-S) Tests. 6

SI-3 Results for CPEs in $NaHCO_3$. 8

SI-4 Results for CPEs of CoMP11-Ac in CAPS, CHES, and MOPS. 10

SI-5 Results of CPE Experiments on CoMP11-Ac in CAPS, CHES, and MOPS. 12

SI-6 Extended (24 hrs) CPE Experiments 15

SI-7 GC Calibration Curves 17

References 19

SI-1 Experimental Details.

Preparation of CoMP11-Ac

CoMP11-Ac was prepared according to a previously reported procedure.[1] The UV-vis spectrum of CoMP11-Ac showed bands with maxima at 416, 528, and 560 nm consistent with previously reported characterization data.[1,2] MALDI-TOF mass spectrometry analysis (using α-cyano-4-hydroxycinnamic acid as matrix) of CoMP11-Ac samples gave peaks between 1949 and 1952 amu which is in agreement with the theoretical mass value of 1949.10 amu.

Cyclic Voltammetry

CV experiments were conducted using a three-electrode setup with a $Ag/AgCl/KCl_{(1M)}$ reference electrode (CH Instruments), a Pt wire counter electrode (surface area ~0.14 cm^2), and a mercury drop electrode (BASi CGME MF-9058 used in static mode) as the working electrode (surface area 2.45×10^{-2} cm^2). All potentials are reported vs. a $Ag/AgCl/KCl_{(1M)}$ reference electrode, calibrated against $K_3Fe(CN)_6/K_2Fe(CN)_6$ showing negligible drift. All voltammograms were collected scanning from 0 V to negative potentials and then back to 0 V. A CH Instruments 620 D potentiostat was used for all electrochemical experiments. CV experiments were conducted with automatic iR compensation enabled in the CHI 620D potentiostat. The uncompensated resistance of the electrochemical circuit is minimized by placing the $Ag/AgCl/KCl_{(1M)}$ reference electrode in close proximity (~5 to 8 mm) to the mercury-drop working electrode.

Samples were prepared by dissolving a small amount of a concentrated stock solution of CoMP11-Ac into the buffer solution to yield the desired concentration of 1 μM CoMP11-Ac in a total volume of 5 mL. The CV working solution was purged with either N_2 for 5 minutes plus CO_2 for 15 minutes or with N_2 for 20 minutes before each experiment. A constant flow of N_2 or CO_2 in the headspace of the CV cell is maintained throughout the experiments. The pH was adjusted for samples under N_2 to match the pH of samples under CO_2 using concentrated HCl. Buffers were prepared in deionized water by dissolving KCl to yield a 0.1 M solution and the desired buffer solid (MOPS, CAPS, or $NaHCO_3$) to yield a 50 mM solution and adjusted to the desired pH with HCl or NaOH.

Dip-and-Stir Test

The dip-and-stir test is a version of the typical rinse test, adapted to a mercury drop electrode.[3] After a single CV scan is collected or the mercury drop is exposed to the catalyst containing solution for one minute, the electrochemical cell is removed, and the counter and reference electrodes are carefully wiped, while the mercury drop remains at the tip of the capillary. The electrodes are then dipped into a new electrochemical cell containing fresh solution with no catalyst. The solution is stirred for 3 minutes using a magnetic stir bar to remove any catalyst-containing droplets from the electrodes and purged with either CO_2 for 5 minutes. A CV is then collected and any above-background activity detected is due to catalyst adsorbed to the mercury drop. More information regarding this adsorption test can be found in reference 3.

Controlled Potential Electrolysis and Product Analysis

Controlled potential electrolysis experiments were performed in a two-compartment cell (H-cell) with a three-electrode system, the two compartments are separate by a P5 glass frit with an average pore size of 1.0-1.6 μm. The working compartment contained the reference ($Ag/AgCl/KCl_{(1M)}$) and working (mercury pool with a surface area of ~ 2.0 cm^2 connected to the circuit by an insulated platinum wire) electrodes. The counter compartment contained a glassy carbon rod counter electrode (surface area ~ 2.5 cm^2). Buffers were prepared in nanopure DI water by dissolving enough solid to make 1 M KCl and 500 mM MOPS, CAPS, or $NaHCO_3$ and adjusted to the desired initial pH with HCl or NaOH. The pH of all MOPS, CAPS, and $NaHCO_3$ solutions after purging with CO_2 was around 6.5 ± 0.1; when purged with N_2 the pH was near 7.2 ± 0.2 for both MOPS and CAPS, and 8.7 ± 0.3 for $NaHCO_3$.

The volumes of the solutions in the counter and working compartments were 6 and 5 mL respectively to account for the space occupied by the mercury pool and to allow for the headspaces in both compartments to be the same. The solutions in both compartments were purged with either a 95:5% CO_2/CH_4 or an 80:20% N_2/CH_4 mixture (from Airgas) before each experiment with the CH_4 serving as an internal standard. The amount of generated H_2 and CO was determined by GC using calibration curves obtained by injecting known volumes of H_2 and CO at 1 atm (Figures S9-S11). The GC instrument is a Shimadzu GC-2014 Fuel Cell Analyzer with thermal conductivity (TCD) and flame ionization (FID) detectors. For each run, at the end of the experiment, headspace from the working compartment was flushed through a 1-mL loop which was injected into the GC.

^{1}H-NMR experiments were conducted to determine whether solution-based products like formic acid or methanol were formed, in no case were these products detected. During the CPE experiment pressure can build up unevenly in the compartments which can cause the solution to move between compartments, in general, these changes in volume were negligible. However, in the case of MOPS-buffered solutions and for the 24-hour CPE experiments that build up significant pressure, steps were taken to mitigate the volume change. In these cases, the CPE cell was modified by connecting both compartments via 2 needles attached to fine tubing of approximately 60 cm in length and 2 mm in inner diameter to help equalize the pressure in both chambers. Different calibration curves were made for the CPE H-cell with connected headspaces (Figures S12 and S13).

TON Calculation

$$\mathbf{TON} = \frac{\mathbf{n_P(X) - n_{P_b}(X)}}{\mathbf{n_T(cat)}}$$

$\mathbf{n_P(X)}$: moles of product X (CO or H_2) detected by GC.

$\mathbf{n_{P_b}(X)}$: moles of product X (CO or H_2) detected by GC after background bulk electrolysis in absence of catalyst.

$\mathbf{n_T(cat)}$: total moles of catalyst in the electrolyte.

FE% Calculation

$$\mathbf{FE\,\%} = \frac{\mathbf{[n_P(X) - n_{P_b}(X)]F\,\nu}}{\mathbf{Q_T - Q_b}} \times \mathbf{100\%}$$

$\mathbf{n_P(X)}$: moles of product X (CO or H_2) detected by GC.

$\mathbf{n_{P_b}(X)}$: moles of product X (CO or H_2) detected by GC after background bulk electrolysis in absence of catalyst.

F: Faraday constant (96,485 C/mol).

ν: number of electrons transferred per molecule of product (2 for both CO and H_2).

$\mathbf{Q_T}$: total charge passed in the CPE experiment.

$\mathbf{Q_b}$: total charge passed in the background CPE experiment in absence of catalyst.

SI-2 Dip-and-Stir (D-S) Tests.

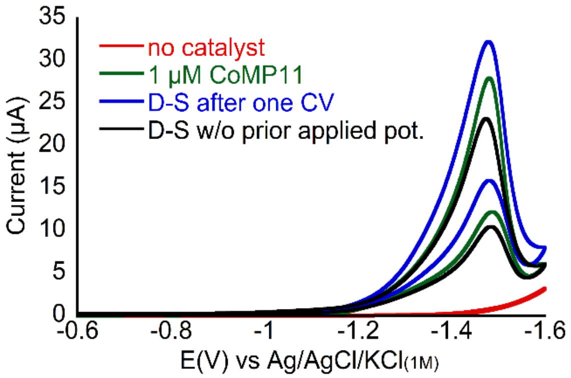

Figure S1. Results of D-S tests performed for 1 μM CoMP11-Ac in 50 mM $NaHCO_3$, 0.1 M KCl, pH 6.2 at 100 mV/s under 1 atm CO_2.

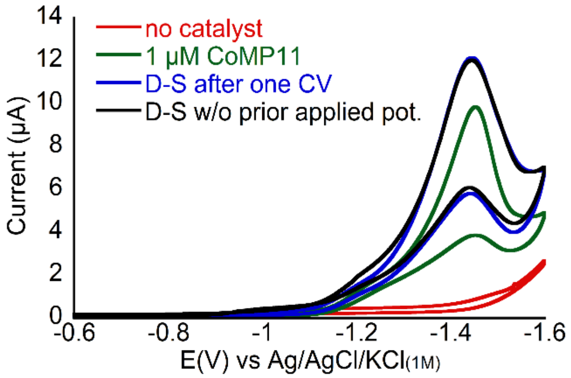

Figure S2. Results of D-S tests performed for 1 μM CoMP11-Ac in 50 mM CAPS, 0.1 M KCl pH 5.5 at 100 mV/s under 1 atm CO_2.

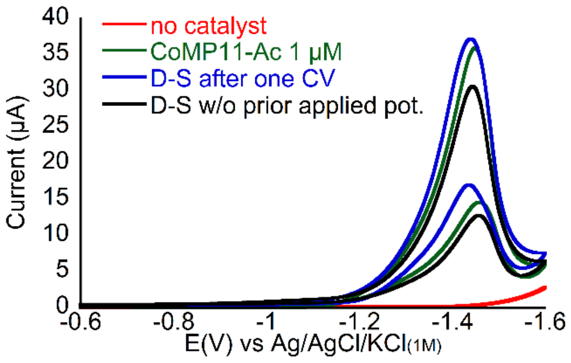

Figure S3. Results of D-S tests performed for 1 μM CoMP11-Ac in 50 mM CHES, 0.1 M KCl pH 5.9 at 100 mV/s under 1 atm CO_2.

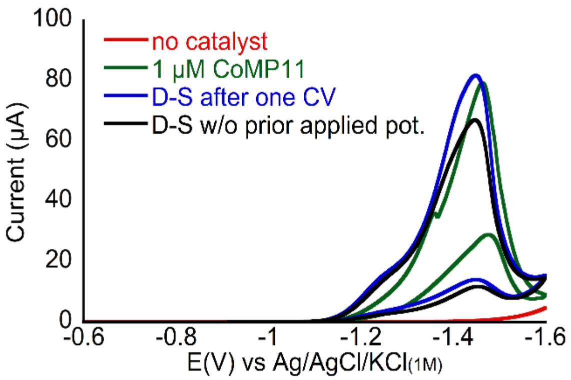

Figure S4. Results of D-S tests performed for 1 μM CoMP11-Ac in 50 mM MOPS, 0.1 M KCl pH 5.9 at 100 mV/s under 1 atm CO_2.

SI-3 Results for CPEs in $NaHCO_3$.

Table S1. Results of 2-hour CPEs of 1 µM CoMP11-Ac in 0.5 M $NaHCO_3$, 1 M KCl.*

	E (V)	FE(H_2)	FE(CO)	TON(H_2)	TON(CO)	Q_T (C)
CO_2	-1.4	25	61	1200	3000	5
		23	57	880	2200	4
		23	65	860	2500	4
	-1.2	4	97	170	4300	4
		4	80	100	2300	3
		3	75	150	3200	4
N_2	-1.4	81	12	1400	220	2
		77	11	1100	160	1
		72	23	940	310	1
	-1.2	No above-background activity**				

* The pH of the $NaHCO_3$ solutions after purging with CO_2 was 6.6 ± 0.1 and 8.7 ± 0.3 when purged with N_2.

** Activity is not reported if the charge was not three times the background level in at least two out of the three replicates.

SI-4 CV at varying CO_2 Partial Pressure.

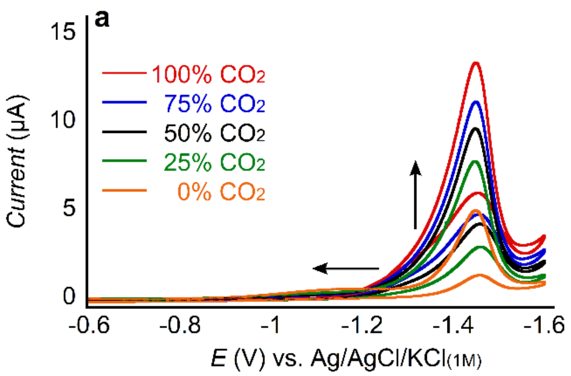

Figure S5. CV of 1 μM CoMP11-Ac in 50 mM CAPS, 0.1 M KCl, pH 6.0 ± 0.2 at 100 mV/s under different P_{CO_2}, the arrows indicate the direction of increasing P_{CO_2}.

SI-5 Results for CPEs of CoMP11-Ac in CAPS, CHES, and MOPS.

Table S2. Results of 2-hour CPEs of 1 µM CoMP11-Ac in 0.5 M CAPS, 1 M KCl.*

	E (V)	FE(H_2)	FE(CO)	TON(H_2)	TON(CO)	Q_T (C)
CO_2	-1.4	34	39	290	340	1
		30	58	281	540	1
		23	46	268	540	1
	-1.2	5	81	77	1200	1
		5	85	99	1700	2
		4	99	72	1700	2
N_2	-1.4	68	0	600	0	1
		99	0	480	0	1
		82	0	420	0	1
	-1.2	No above-background activity**				

* The pH of the CAPS solutions after purging with CO_2 was 6.6 ± 0.1 and 7.1 ± 0.3 when purged with N_2.

** Activity is not reported if the charge was not three times the background level in at least two out of the three replicates.

Table S3. Results of 2-hour CPEs of 1 µM CoMP11-Ac in 0.5 M CHES, 1 M KCl

	E (V)	FE(H_2)	FE(CO)	TON(H_2)	TON(CO)	Q_T (C)
CO_2	-1.4	37	60	950	1600	2.5
		51	58	969	1100	1.8
		41	53	910	1200	2.2
	-1.2	7	81	280	3200	3.9
		5	79	240	3800	4.6
		6	82	240	3500	4.1
N_2	-1.4	100	0	3700	0	3.6
		81	0	2900	0	3.5
		96	0	1800	0	1.8
	-1.2	73	2	650	4	0.9
		67	0	630	0	0.9
		63	0	490	0	0.8

* The pH of the CHES solutions after purging with CO_2 was 6.6 ± 0.1 and 7.1 ± 0.3 when purged with N_2.

Table S4. Results of 2-hour CPEs of 1 μM CoMP11-Ac in 0.5 M MOPS, 1 M KCl.*

	**E (V)**	**FE(H_2)**	**FE(CO)**	**TON(H_2)**	**TON(CO)**	**Q_T (C)**
**CO_2**	-1.4	74	16	4300	940	6
		66	21	4300	1400	6
		50	26	3600	1900	7
	-1.2	8	83	1200	13000	15
		7	84	1100	13000	15
		10	87	1300	11000	13
**N_2**	-1.4	96	0	20000	0	21
		95	0	24000	0	25
		84	0	26000	0	30
	-1.2	99	1	4200	48	4
		100	2	5900	79	5
		95	1	4700	66	5

* The pH of the MOPS solutions after purging with CO_2 was 6.6 ± 0.1 and 7.1 ± 0.3 when purged with N_2.

SI-6 Results of CPE Experiments on CoMP11-Ac in CAPS, CHES, and MOPS.

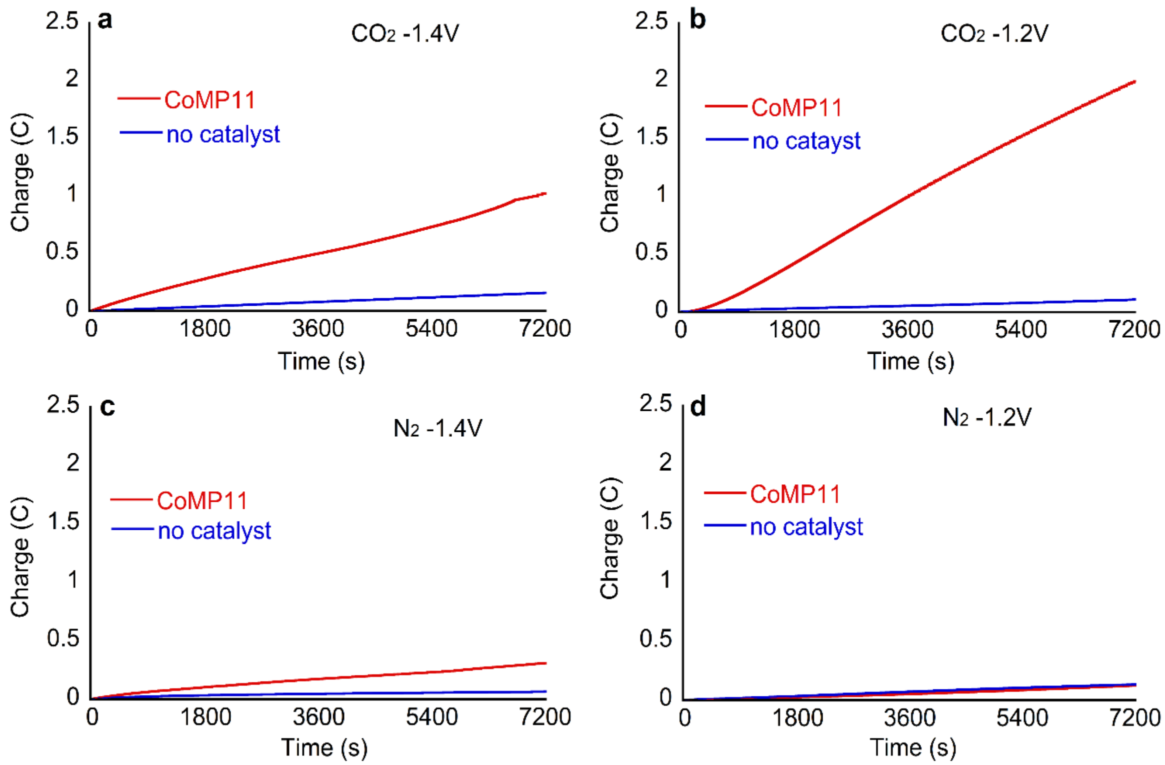

Figure S6. CPE experiments run in 0.5 M CAPS, 1 M KCl, the concentration of catalyst was 1 μM when present. CPE run in **(a)** CO_2-saturated solution at -1.4 V, **(b)** CO_2-saturated solution at -1.2 V, **(c)** N_2-saturated solution at -1.4 V, and **(d)** N_2-saturated solution at -1.2 V. pH = 6.6 ± 0.1 for **(a)** and **(b),** and 7.2 ± 0.2 for **(c)** and **(d)**. Potentials reported vs. Ag/AgCl/$KCl_{(1M)}$.

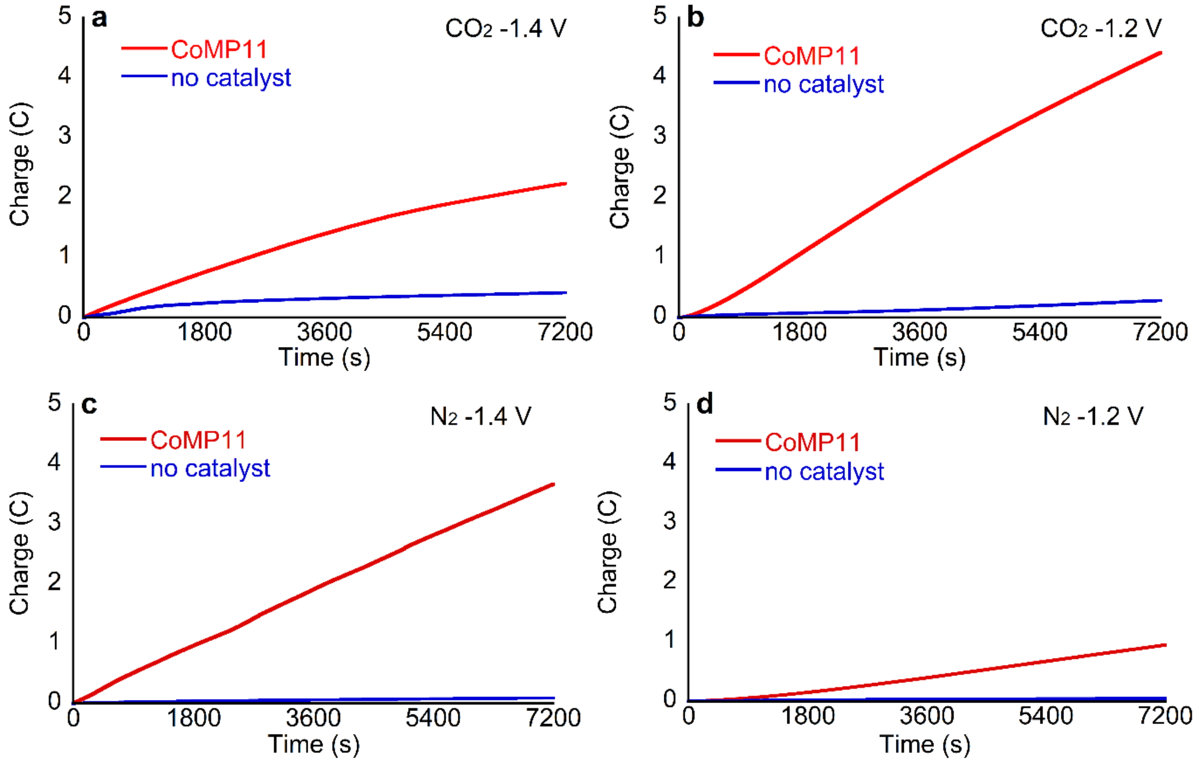

Figure S7. CPE experiments run in 0.5 M CHES, 1 M KCl, the concentration of catalyst was 1 µM when present. CPE run in **(a)** CO_2-saturated solution at -1.4 V, **(b)** CO_2-saturated solution at -1.2 V, **(c)** N_2-saturated solution at -1.4 V, and **(d)** N_2-saturated solution at -1.2 V. pH = 6.6 ± 0.1 for **(a)** and **(b),** and 7.1 ± 0.3 for **(c)** and **(d)**. Potentials reported vs. Ag/AgCl/$KCl_{(1M)}$.

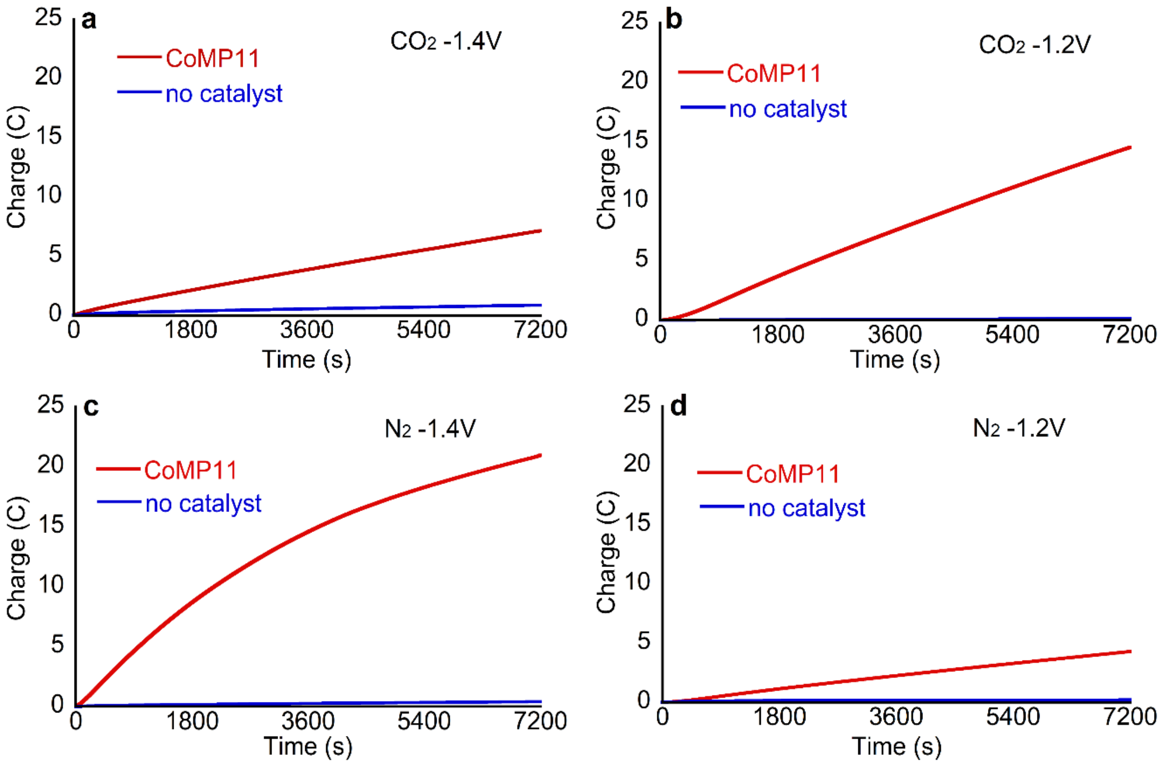

Figure S8. CPE experiments run in 0.5 M MOPS, 1 M KCl, the concentration of catalyst was 1 μM when present. CPE run in **(a)** CO_2-saturated solution at -1.4 V, **(b)** CO_2-saturated solution at -1.2 V, **(c)** N_2-saturated solution at -1.4 V, and **(d)** N_2-saturated solution at -1.2 V. pH = 6.6 ± 0.1 for **(a)** and **(b),** and 7.2 ± 0.2 for **(c)** and **(d)**. Potentials reported vs. Ag/AgCl/$KCl_{(1M)}$.

SI-7 Extended (24 hrs) CPE Experiments

Table S5. Results of 24-hour CPEs of 1 µM CoMP11-Ac in 0.5 M buffer, 1 M KCl under CO_2.*

Buffer	E (V)	FE(H_2)	FE(CO)	TON(H_2)	TON(CO)	Q_T (C)
$NaHCO_3$	-1.2	6 ± 5	76 ± 8	680 ± 510	9100 ± 1200	12 ± 3
CAPS	-1.2	5 ± 4	85 ± 14	560 ± 490	9300 ± 500	11 ± 2
CAPS	-1.4	11 ± 6	58 ± 6	800 ± 420	4500 ± 1900	7.4 ± 2
CHES	-1.2	12 ± 6	72 ± 12	2800 ± 700	16000 ± 600	22 ± 3
MOPS	-1.2	9 ± 2	61 ± 15	4600 ± 260	32000 ± 8900	51 ± 7

* The pH of all MOPS, CAPS, CHES and $NaHCO_3$ solutions after purging with CO_2 was 6.6 ± 0.1.

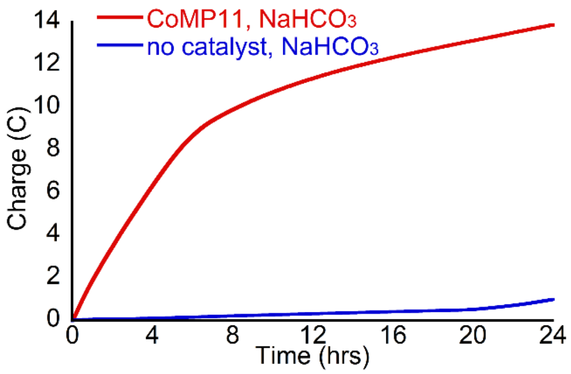

Figure S9. CPE experiments run in 0.5 M $NaHCO_3$, 1 M KCl, under CO_2 at -1.2 V, pH = 6.6 ± 0.1, the concentration of catalyst was 1 µM when present.

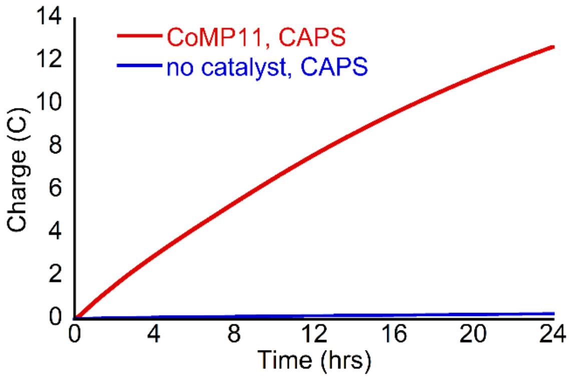

Figure S10. CPE experiments run in 0.5 M CAPS, 1 M KCl, under CO_2 at -1.2 V, pH = 6.6 ± 0.1, the concentration of catalyst was 1 µM when present.

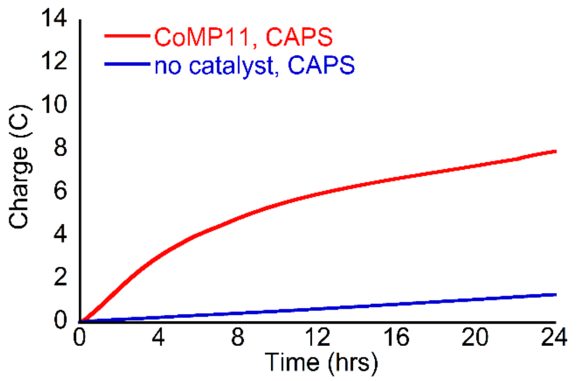

Figure S11. CPE experiments run in 0.5 M CAPS, 1 M KCl, under CO_2 at -1.4 V, pH = 6.6 ± 0.1, the concentration of catalyst was 1 μM when present.

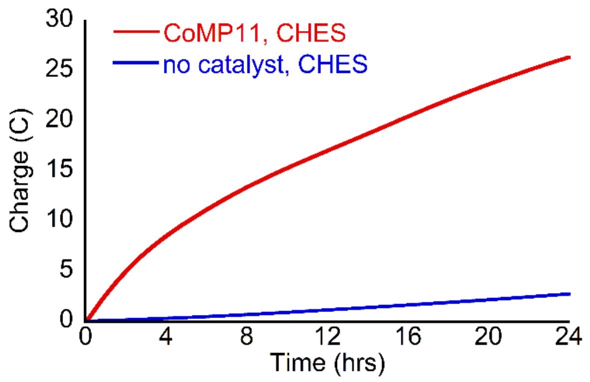

Figure S12. CPE experiments run in 0.5 M CHES, 1 M KCl, under CO_2 at -1.2 V, pH = 6.6 ± 0.1, the concentration of catalyst was 1 μM when present.

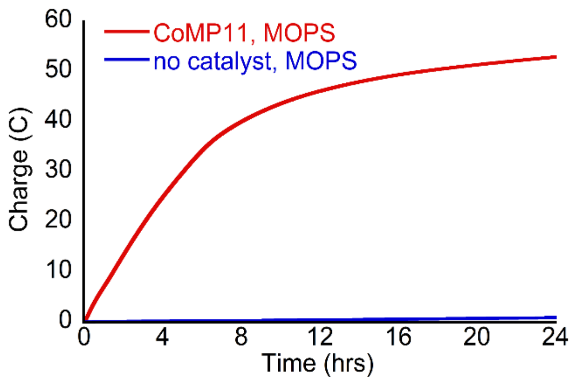

Figure S13. CPE experiments run in 0.5 M MOPS, 1 M KCl, under CO_2 at -1.2 V, pH = 6.6 ± 0.1, the concentration of catalyst was 1 μM when present.

SI-8 GC Calibration Curves

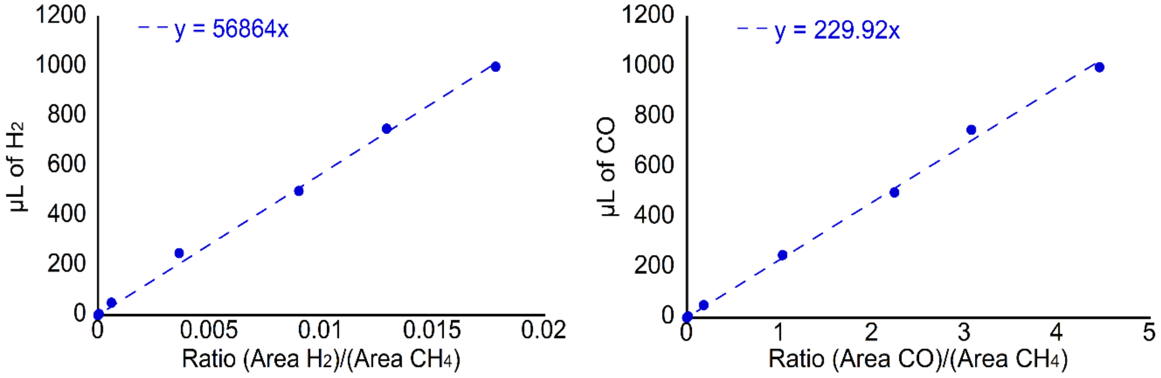

Figure S14. Calibration curves for the quantification of H_2 (left) and CO (right), with 95:5% CO_2:CH_4 as the purging gas.

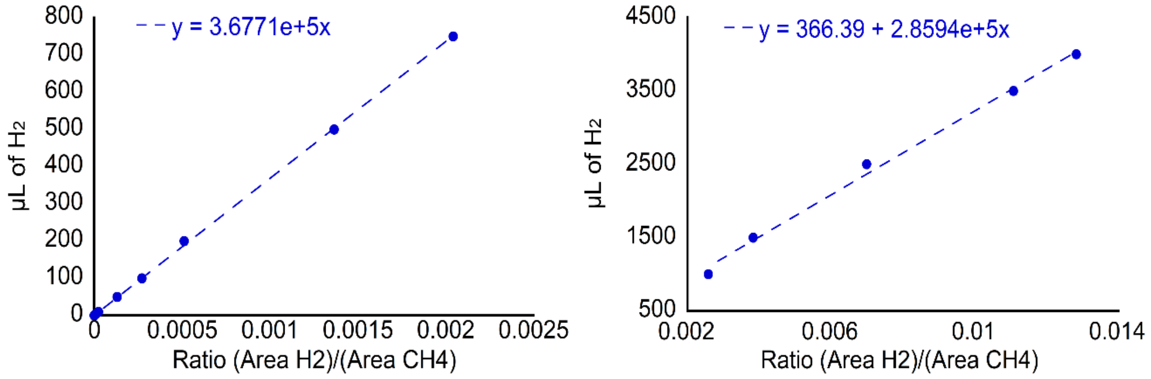

Figure S15. Calibration curves for the quantification of H_2 with 80:20% N_2:CH_4 as the purging gas. (Left) Low Volumes. (Right) High Volumes.

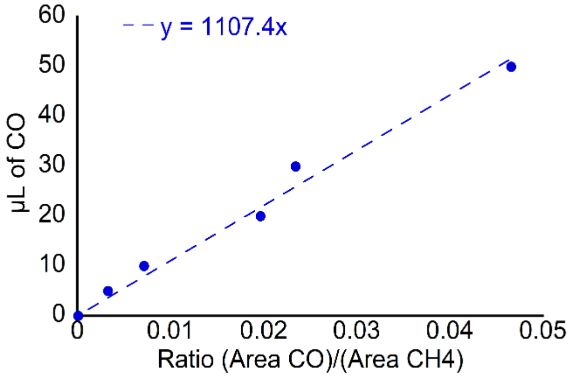

Figure S16. Calibration curve for the quantification of CO with 80:20% N_2:CH_4 as the purging gas.

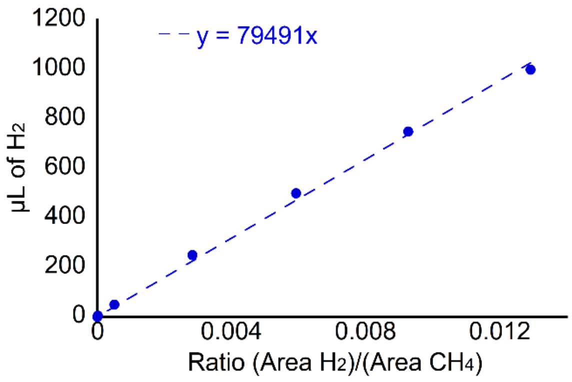

Figure S17. Calibration curve for the quantification of H_2 with 95:5% CO_2:CH_4 as the purging gas in an H-cell with connected headspaces.

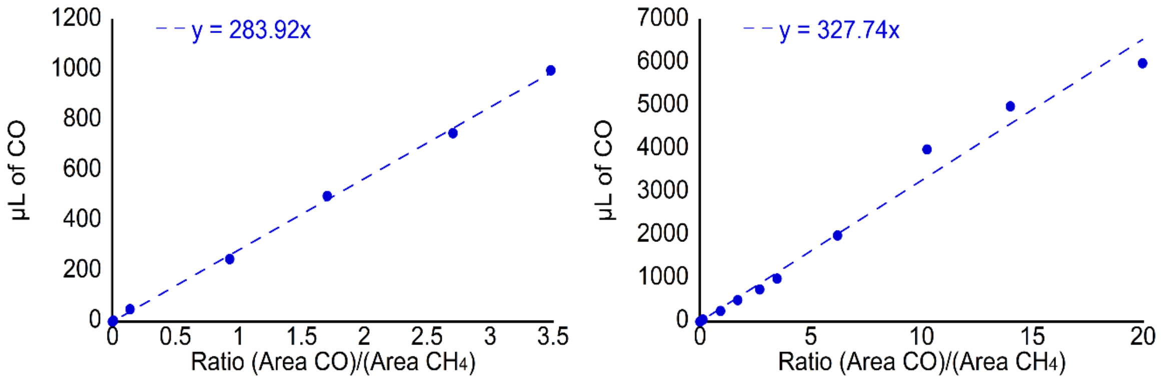

Figure S18. Calibration curves for the quantification of CO with 95:5% CO_2:CH_4 as the purging gas in an H-cell with connected headspaces. (Left) Low volumes. (Right) High volumes.

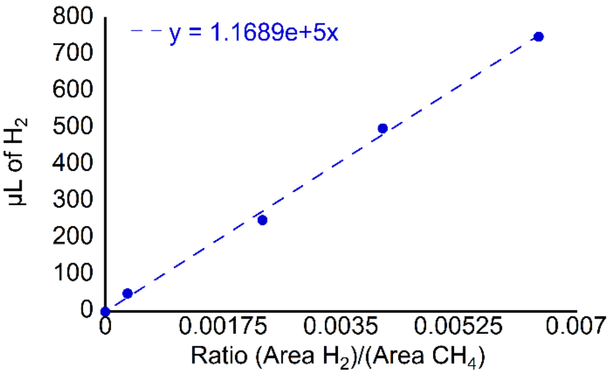

Figure S19. Calibration curve for the quantification of H_2 with 95:5% CO_2:CH_4 as the purging gas in an H-cell with connected headspaces after instrument repairs.

References

1. Kleingardner, J. G.; Kandemir, B.; Bren, K. L. Hydrogen Evolution from Neutral Water under Aerobic Conditions Catalyzed by Cobalt Microperoxidase-11. *J. Am. Chem. Soc.* , **2014**, 136, 4-7.
2. Alvarez-Hernandez, J. L.; Sopchak, A. E.; Bren, K. L. Buffer pKa Impacts the Mechanism of Hydrogen Evolution Catalyzed by a Cobalt Porphyrin-Peptide. *Inorg. Chem.*, **2020**, 59, 8061-8069.
3. Alvarez-Hernandez, J. L.; Han, J. W.; Sopchak, A. E.; Guo, Y.; Bren, K. L. Linear Free Energy Relationships in Hydrogen Evolution Catalysis by a Cobalt Tripeptide in Water. *ACS Energy Lett.*, **2021**, 6, 2256-2261.
